# Coital resumption after delivery among OASIS patients: differences between instrumental and spontaneous delivery

**DOI:** 10.1186/s12905-019-0845-8

**Published:** 2019-12-06

**Authors:** Sònia Anglès-Acedo, Cristina Ros-Cerro, Sílvia Escura-Sancho, Núria Elías-Santo-Domingo, M. José Palau-Pascual, Montserrat Espuña-Pons

**Affiliations:** Pelvic Floor Unit, ICGON, Hospital Clínic de Barcelona, University of Barcelona, Villarroel 170, 08036 Barcelona, Spain

**Keywords:** Mode of delivery, OASIS, Perineal trauma, Coital resumption, Female sexual function, Vaginal delivery

## Abstract

**Background:**

Obstetric anal sphincter injuries (OASIS) are associated with sexual dysfunction and a lower likelihood of sexual activity in the postpartum period. The aim of the present study was to compare coital resumption and the variables influencing this activity after delivery in women with and without a history of obstetric anal sphincter injury (OASIS) and according to the mode of delivery.

**Methods:**

A prospective, observational, case-control study was performed at 6 months postpartum in 318 women: 140 with a history of primary repaired OASIS and 178 women without OASIS. Demographic and obstetric data, breastfeeding, and symptoms of urinary and anal incontinence were collected. Patients were asked about coital resumption and completed the validated specific Pelvic Organ Prolapse/Urinary Incontinence Sexual Questionnaire-12 (PISQ-12). Continuous and non-continuous variables were compared using ANOVA and the Fisher exact tests, respectively. A multivariate logistic regression model and a multiple regression analysis were constructed to assess the impact of demographic and clinical variables on the percentage of coital resumption and on the PISQ-12 score, respectively.

**Results:**

After a spontaneous delivery (SD), patients without OASIS showed a higher percentage of coital resumption than those with OASIS (98% vs. 77%; *p* = 0.003), and the PISQ-12 score was also higher (*p* < 0.001). PISQ-12 score was better in women with SD compared to those with operative vaginal delivery (OVD)(*p* < 0.001), independently of the history of OASIS. Current breastfeeding, a higher Wexner score and OVD negatively influenced the PISQ-12 score.

**Conclusions:**

After SD, women with OASIS resumed coital activity later than women without OASIS. Women with OVD resumed coital activity later, and had a lower PISQ-12 score than women with SD.

## Background

Obstetric anal sphincter injuries (OASIS) involve severe perineal injury with anatomic and functional consequences, which can affect sexual activity and function. OASIS seems to be an independent risk factor for sexual dysfunction and postponed intercourse resumption after delivery [1, 2]. The cause of this delay and the lower quality of sexuality among these patients is under-explored. However, knowledge on sexual complaints and time to sexual resumption after OASIS is scarce.

Some authors conclude that the severity of anal incontinence (AI) is related to sexual dysfunction [3], while others consider perineal trauma as the factor having the greatest affect on sexual function on comparing women with OASIS with women with less severe perineal injury [1]. Other variables such as maternal age, current breastfeeding or symptoms of pelvic floor dysfunction have also previously been described as factors influencing the resumption of coitus after delivery [1].

On the other hand, regardless of the history of OASIS, operative vaginal delivery (OVD) has been associated with poorer sexual functioning [4] in comparison with spontaneous delivery (SD) or caesarean section (CS).

In this context, the aim of the present study was to investigate coital resumption and the variables influencing this activity at 6 months postpartum in patients with and without OASIS analysed according to the mode of delivery.

## Methods

### Participants and procedures

An observational, cross sectional, case-control study was designed and conducted in a tertiary university hospital from September 2012 to June 2017. Women in whom OASIS was identified and repaired intrapartum were selected for the study group, and women without a history of OASIS were selected for the control group. All women were recruited in the delivery room immediately postpartum. Delivery was vaginal in all the participants, being either by SD or OVD (including Naegele and Kjelland forceps, Thierry’s spatulas and vacuum). The exclusion criteria included patients with a history of OASIS in previous deliveries, twins, delivery by CS or the inability to comprehend questionnaires. All the women included were assessed at a single visit at 6 months after delivery.

The study was approved by the Ethical Committee of our hospital and written informed consent was obtained from all the participants. All methods were performed in accordance with relevant guidelines and regulations.

### Materials

The main outcome was coital resumption and sexual function at 6 months postpartum. All women completed the validated self-administered Pelvic Organ Prolapse/Urinary Incontinence Sexual Questionnaire-12 (PISQ-12) [5, 6]. The PISQ-12 is the short-version of a condition-specific questionnaire to target women with pelvic floor disorders (urinary incontinence and pelvic organ prolapse). Considering the high rate of pelvic floor symptoms during the postpartum period [7], a condition-specific questionnaire was chosen for the present study. Responses are graded on a 5-point Likert scale from ‘never’ to ‘always’ and the total score ranges from 0 to 48. Questions 1–4 are scored from 0 to 5, and the rest are scored reversely. Higher scores reflect better sexual function and lower scores poorer sexual function [5].

Two more validated questionnaires were self-administered: the Wexner’s score to evaluate the severity of AI symptoms, ranging from 0 to 20 [8], and the International Consultation on Incontinence Questionnaire (ICIQ-UI-SF) was used to detect symptoms and severity of urinary incontinence (UI) (score 0–21) [9]. In both questionnaires higher scores reflect poorer function.

Demographic data, obstetric history and current breastfeeding were collected.

Two expert urogynaecologists performed a gynaecological examination and assessed pelvic floor muscle strength, by digital vaginal palpation, according to the Modified Oxford Grading Scale (MOS) (range 0–5).

Intrapartum identification and repair of OASIS was performed by a senior obstetrician according to the guidelines of the Royal College of Obstetricians and Gynaecologists [10]. All the participants underwent 3D-endoanal ultrasound at 6 months pospartum to evaluate the anatomy of the anal sphincter complex to detect the presence of residual tears after primary repair (OASIS group) or the presence of occult OASIS (non-OASIS group). Women with undiagnosed intrapartum OASIS were also excluded. 3D volumes were obtained using a 360° mechanical rotational probe with automatic 3D acquisition (type 2052, Ultraview-800 BK-Medical), at a frequency of 13 MHz [11] and assessed by an expert.

### Sample size calculation

The sample size of the study was not originally calculated considering the main outcome of the present investigation. A post-hoc analysis showed that the sample of women was large enough to detect between-group differences of 15% of coital resumption and the usual α (0.05) and β levels (80%).

### Statistical analysis

The statistical analysis was performed with the SPSS software package (19.0 version, SPSS Inc., Chicago, IL). Continuous variables were compared between groups using ANOVA tests with Bonferroni correction when the analysis was performed with more than two groups. Comparison of non-continuous variables was performed using the Fisher exact test.

A multivariate logistic regression model was constructed to assess the impact of demographic and clinical variables (age at delivery, mode of delivery, mediolateral episiotomy, history of OASIS, multiparity, breastfeeding, ICIQ-UI-SF score, the Wexner score and MOS) on the percentage of coital resumption. In addition, a multiple regression analysis was undertaken with the same variables to assess their impact on the PISQ-12 score. Variables with a *p* value less than 0.050 in the analysis were included in the multiple regression model, and those with tolerance to collinearity less than 0.1 were excluded.

## Results

### Characteristics of the participants

Of the 331 women originally recruited, 13 were excluded because of undiagnosed intrapartum OASIS. Thus, a total of 318 women were included in the study: 140 women with a history of OASIS and 178 women without OASIS. The length of time between delivery and the study visit was 183 ± 69 days, without differences between groups. Among the OASIS cases, 67 (48%) were classified intrapartum as 3A, 60 (43%) as 3B, 7 (5%) as 3C and 6 (4%) as 4. Of the 215 OVD, 59% were Kjelland forceps, 21% Naegele forceps, 7% Thierry’s spatulas and 13% vacuum extraction.

Table 1 shows the demographic data, the obstetric history, MOS score, the ICIQ-UI-SF and Wexner scores of all the participants, according to the history of OASIS and classified by the mode of delivery (SD vs. OVD).

**Table 1:** Demographic data, obstetric history and pelvic floor characteristics according to the history of obstetric anal sphincter injuries (OASIS) and classified by the mode of delivery: spontaneous delivery (SD) and operative vaginal delivery (OVD)

	Non-OASIS	OASIS	*p*
Maternal age at delivery	33.7 ± 4.5	33.2 ± 5.2	NS
SD	34.0 ± 4.0	33.2 ± 4.5	NS
OVD	33.5 ± 4.6	33.2 ± 5.8	NS
Gestational age	40.0 ± 1.3	40.0 ± 1.3	NS
SD	39.8 ± 1.3	40.1 ± 1.1	NS
OVD	40.1 ± 1.3	39.9 ± 1.4	NS
Newborn weight	3318 ± 428	3431 ± 414^1^	*p* = 0.02
SD	3227 ± 512	3460 ± 370^2^	*p* = 0.04
OVD	3346 ± 389	3409 ± 446	NS
Mediolateral episiotomy	131/178 (74%)	62/139^3^ (45%)	*p* < 0.0001
SD	11/43 (26%)	15/60 (25%)	NS
OVD	120/135 (89%)	47/79 (59%)^4^	p < 0.0001
Breastfeeding	143/169 (85%)	87/135 (64%)	NS
SD	27/35 (77%)	42/58 (72%)	NS
OVD	102/134 (76%)	59/77 (77%)	NS
Previous vaginal deliveries	26/178 (15%)	19/132 (15%)	NS
SD	20/43 (47%)	14/58 (24%)	NS
OVD	6/135 (4%)	5/74 (7%)	NS
ICIQ-UI-SF score	2.6 ± 0.3	2.6 ± 0.4	NS
SD	2.7 ± 0.6	2.6 ± 0.6	NS
OVD	2.5 ± 0.4	2.5 ± 0.5	NS
ICIQ-UI-SF > 0	64/178 (36%)	45/140 (32%)	NS
Wexner score	1.1 ± 1.6	2.7 ± 3.2	0.001
SD	1.2 ± 1.8	2.5 ± 3.3	0.02
OVD	1.1 ± 1.6	2.9 ± 3.2	0.001
Wexner> 0 N (%)	82/178 (46%)	93/140 (66%)	< 0.001
SD	22/43 (51%)	39/60 (65%)	0.001
OVD	60/135 (44%)	54/80 (68%)	0.001
Modified Oxford Scale	2.6 ± 0.1	2.1 ± 0.1	0.02
SD	2.9 ± 0.5	2.4 ± 0.23	0.04
OVD	2.6 ± 0.2	1.8 ± 0.1	0.001

### Postpartum sexual activity and function

Globally, the percentage of patients with coital resumption at 6 months was 73%, without statistically significant differences between the OASIS vs. non-OASIS groups (Table 2).

**Table 2:** Coital resumption and sexual function at 6 months postpartum, according to the history of obstetric anal sphincter injuries (OASIS) and classified by the mode of delivery: spontaneous delivery (SD) and operative vaginal delivery (OVD)

	Non-OASIS	OASIS	Total
Coital resumption	123/178 (69%)	108/140 (77%)	231/318 (73%)
SD	42/43 (98%)^2,3^	46/60 (77%)^3^	88/103 (85%)^1^
OVD	81/135 (60%)^2,4^	62/80 (78%)^4^	143/215 (67%)^1^
PISQ-12 score	32.5 ± 9.1	34.1 ± 7.3	34.2 ± 6.7
SD	39.7 ± 5.4^5^	33.8 ± 7.3^5,^	36.9 ± 7.0^6^
OVD	31.6 ± 4.4^7^	39.9 ± 1.4^7^	32.5 ± 5.9^6^

^1^ SD vs. OVD: *p* = < 0.0001; ^2^SD vs. OVD among patients without OASIS: *p* = < 0.0001; ^3^OASIS vs. non-OASIS among SD deliveries: *p* = 0.003; ^4^OASIS vs. non-OASIS among OVD deliveries: *p* = 0.01

^5^Among SD, OASIS vs. non-OASIS (*p* < 0.001); ^6^ SD vs. OVD (*p* = < 0.001); ^7^Among OVD, OASIS vs. non-OASIS (*p* = 0.03)

However, important differences were observed when the delivery mode was considered (Table 2). Among women without OASIS who had a SD, 98% resumed coital activity at 6 months postpartum. Surprisingly, after an OVD, the OASIS group seemed to resume coital activity before the non-OASIS group (60% vs. 78%; respectively; *p* = 0.01).

Overall, the total PISQ-12 score at 6 months postpartum between patients with and without OASIS was similar. Nevertheless, when the analysis was made according to the delivery mode, the highest PISQ-12 score was achieved in patients with SD without OASIS (*p* < 0.001) (Table 2). After an OVD, the PISQ-12 score in the OASIS group was better than that in the non-OASIS group (*p* = 0.03). These results were confirmed comparing SD and OVD independently of the history of OASIS, observing that women with SD showed better PISQ-12 scores than those with OVD (*p* < 0.001).

Finally, the influence of demographic and clinical variables (age at delivery, mode of delivery, mediolateral episiotomy, history of OASIS, multiparity, breastfeeding, ICIQ-UI-SF score, the Wexner score and MOS) on coital resumption and the PISQ-12 score were analysed by multivariate tests. The multivariate logistic regression model showed that only maternal age at delivery obtained a statistically significant odds ratio in the percentage of coital resumption, with older women showing the lowest percentage (Table 3). Multiple regression analysis showed that the Wexner score, a history of OVD compared to SD and current breastfeeding at 6 months had a negative influence on the PISQ-12 score.

**Table 3:** Multivariate logistic regression model evaluating the influence of different variables on sexual activity (yes/no) at 6 months postpartum. OVD: operative vaginal delivery; ICIQ-UI-SF: International Consultation on Incontinence Questionnaire; obstetric anal sphincter injuries (OASIS). Multiple regression analysis with the same variables, to assess the impact on the PISQ-12 score

Outcomes	Logistic regression Odds-Ratio	Multiple regression analysis standardized coefficient
OVD	0.70 (0.29–1.71)	−0.142 (*p* = 0.049)
Oxford	0.92 (0.74–1.14)	0.053 (*p* = 0.496)
Breastfeeding	0.63 (0.27–1.45)	−0.227 (*p* = 0.002)
ICIQ-UI-SF	1.03 (0.96–1.18)	−0.061 (*p* = 0.451)
Wexner	0.94 (0.83–1.06)	−0.216 (p = 0.002)
Age at delivery	0.92 (0.86–0.99)^1^	−0.112 (*p* = 0.157)
OASIS	1.28 (0.59–2.78)	−0.007 (*p* = 0.937)
Mediolateral episiotomy	1.86 (0.87–3.98)	0.021 (*p* = 0.801)
Previous vaginal deliveries	0.99 (0.39–2.55)	0.008 (*p* = 0.919)

^1^*p* < 0.05

## Discussion

The main findings of the present study show that after SD, coital resumption at 6 months was lower in the OASIS group than in non-OASIS group. Globally, women with OVD resumed coital activity later and had worse sexual function than women with SD.

Obstetric outcomes can contribute to perineal and pelvic trauma and may have an impact on sexual activity. In this respect, we found that the percentage of mediolateral episiotomy was lower in the OASIS group compared to the non-OASIS group, suggesting that the use of mediolateral episiotomy is associated with a reduction in the rate of OASIS. Recent studies have reported that the use of mediolateral episiotomy during OVD reduces the rate of OASIS five- to ten-fold in primiparous and multiparous women [12, 13]. Additionally, newborn weight is also considered a risk factor for OASIS [7, 14], and thus, women with OASIS delivered larger newborns than those without OASIS. Finally, the low rate of multiparous in the group of OVD and OASIS in the present study supports the contention that the first vaginal delivery which requires instrumentation causes the most important pelvic floor damage [15].

Several recent studies have described the time to coital resumption after delivery among patients with OASIS [1–3]. In a cohort of women with OASIS only 40% admitted coital resumption at 3 months postpartum [2], being much lower than the 77% reported at 6 months in our study. The longer follow-up to 6 months could explain these differences, considering the previous report of a gradual improvement of perineal pain from 2 to 6 months after delivery [16]. The previously mentioned cohort study [2] did not compare patients according to the delivery method, and neither was a control group included. Conversely, Fodstad [1] compared the sexual function of women with OASIS and controls and found that up to 75.2% of women resumed coital activity at 3 months after delivery, similar to our global 73% at 6 months postpartum. Considering the time to coital resumption after delivery and different modes of delivery, no significant difference in the percentage of distribution of coital resumption was found in the Fodstad study [1], probably because the 13% of OVD in their sample were performed exclusively by vacuum, which is not as damaging to the perineum as forceps delivery [17]. In contrast, we found a higher percentage of coital resumption among women after SD compared to those after OVD, probably because forceps were the main instrument used in our sample (80%), which seems to be more harmful [14].

OASIS has been associated with more perineal pain and dyspareunia compared with other perineal trauma, even if properly repaired [5, 7]. OASIS was a strong predictor of postponed coital resumption in the Fodstad study [1]. Women with OASIS and more severe AI symptoms reported worse sexual function after return to coitus [2]. In fact, independently of OASIS, AI symptoms have also been associated with sexual complaints [18, 19]. In the present study, the Wexner scores were found to influence the PISQ-12 scores in the multiple regression model. The perineal damage causing AI symptoms due to OASIS or OVD without OASIS [16] seemed to have a greater influence on sexual function than residual tears of the anal sphincter complex. In fact, our data showed that OVD with forceps might be responsible for further pelvic damage and worse sexual function in addition to OASIS. In a prospective, multicentre, cohort study, McDonald et al. [20] performed a multivariable logistic regression model to obtain a more accurate association between dyspareunia and delivery mode. The results showed that women with OVD (with forceps or vacuum extraction) had a greater than three-fold increase in the adjusted odds ratio of dyspareunia at 6 months postpartum [20].

The impact of postpartum UI and AI on sexual activity is generally accepted and, in our study, the ICIQ-UI-SF results did not suggest any relationship between postpartum UI and OASIS or delivery mode. Scheer*et al* [21]. reported a higher rate of UI among women with OASIS compared to women without OASIS (38% vs. 21.2%), while other authors do not support these differences [7]. In the Scheer study [21] UI symptoms were evaluated at 10 weeks, while in the present study UI symptoms were evaluated at 6-months, a time at which the tissue of the pelvic floor is more likely to have recovered and remodelled.

Our results regarding AI are in concordance with the literature, with the OASIS group showing worse results in Wexner scores, as well as a high percentage of AI symptoms in comparison with women without OASIS. Our percentages of AI symptoms were very high, due to the large proportion of OVD in the sample (67.6%). Nonetheless, this was a case-control study which allowed the comparison of groups, but not the calculation of prevalences. However, the high proportion of women referring AI symptoms in the non-OASIS group in our study was of note.

It should be taken into account that perineal tear is only one physical factor, and there are other factors that likely influence the decision of women to be sexually active in the postpartum period. In the present study, one quarter of the women (27%) were sexually inactive at 6 months postpartum, independently of the history of OASIS and delivery mode. These results suggest that other factors such as fatigue, partner sexuality or body and genital self-image, likely influence the decision of women to resume coital activity [22]. Similar to the results of the multivariate model in the Fodstad study [1], in the present study, maternal age influenced the time to coital resumption. Moreover, Barbara et al. [4] found that women with current breastfeeding postponed the onset of coitus, having poorer scores in sexual questionnaires. We also found that current breastfeeding at 6 months postpartum had a negative influence on sexual function. These results suggest the role of hormonal status and other subjective factors in postpartum sexual function [4, 16].

Independently of the history of OASIS and delivery mode, the mean PISQ-12 score among all the women who resumed coital activity at 6 months postpartum was 34.2 out of a possible score of 48, being lower than that of other populations not involving the postpartum period. Accordingly, our findings indicate that women reported a significant reduction in sexual function at 6 months postpartum, based on the results of the Spanish Validation study of the PISQ-12, which showed a mean total score of 36.8 among women with pelvic organ prolapse and/or UI. According to the Women’s Sexual Function questionnaire [6], this value is correlated with the presence of a sexual function disorder.

Despite the high prevalence of symptoms of pelvic floor dysfunction at 6 months postpartum, including sexual disorders, women often avoid seeking professional help due to embarrassment. Only 5.6% of women with AI refer these symptoms to a general practitioner and 15.9% to an urogynaecologist [23]. On the other hand, three quarters of the urogynaecologists did not enquire about symptoms of pelvic floor dysfunction postnatally [24]. Surprisingly, the need for help with sexual health problems was expressed by only 7 to 13% of women probably due to stigma and unfamiliarity with treatment options [22, 25]. The results of the present study at 6 months postpartum suggest the potential benefit of extending postpartum follow-up visits beyond the typical 6–8 weeks in order to evaluate the persistence of symptoms of pelvic floor dysfunction [16]. In addition to evaluating UI and AI symptoms, the simple question of *“Have you resumed coital activity?”* could be a screening tool to evaluate the impact of perineal trauma, the treatable scars and painful areas causing dyspareunia and their possible association with a delay in coital resumption.

The main strengths of this study are the large sample size and the detailed analysis according to the mode of delivery, including a great number of forceps deliveries. The follow-up at 6 months postpartum differs from the short-term evaluation performed in most of the previously published studies and provides new data. A post-hoc analysis showed that the sample size was large enough, although it was not originally calculated considering the main outcome of the present investigation. The study has several limitations. On being a case-control study it was not possible to calculate the prevalence of symptoms in the sub-groups of patients, and the cross-sectional design did not allow obtaining information on potential sexual dysfunction and mental health disorders before and during pregnancy or in relation to other social and psychological factors. We are aware that the PISQ-12 is a condition-specific questionnaire that has been validated to assess urinary incontinence and pelvic organ prolapse, but it is not specific for anal incontinence, although there is one question related to sexual activity restriction due to fear of stool or urinary incontinence. However, when we started the present study, the PISQ-12 was the only condition-specific questionnaire to target women with pelvic floor disorders that was validated in Spanish. Nonetheless, it is likely that at present the Pelvic Organ Prolapse/Urinary Incontinence Sexual Questionnaire- IUGA Revised, which was validated in Spanish in 2017, would be used. Moreover, the addition of the Female Sexual Function Index or a semistructured face-to-face interview, rather than a single measurement of sexual functioning with PISQ-12, would have provided a stronger and more comprehensive assessment of sexual functioning. In addition, data related to OVD were analysed including all the instruments despite the controversy about the role of vacuum in pelvic floor damage [17]. Unfortunately, the low number of vacuum deliveries in the present sample did not allow sufficient power to perform a sub-analysis. The lack of a group of women with CS in the control group could also be interpreted as a limitation.

## Conclusions

In conclusion, a history of OASIS delays the resumption of coital activity among women with SD. However, a history of OVD seems to have a greater impact on that decision, thereby reflecting the long-term effects of severe perineal and pelvic trauma on postpartum sexuality. Although our data focused on physical and hormonal factors, this study provides evidence that at mid-term postpartum a high percentage of women, especially those with perineal and pelvic trauma, still continue to have pelvic floor and sexual disorders.

Therefore, the extension of postpartum follow-up visits beyond the typical 6–8 weeks could imply potential benefits in order to evaluate the persistence of these symptoms. Other factors such as maternal age, current breastfeeding or AI symptoms should also be evaluated when assessing postpartum sexual function. A short and direct question such as *“¿Have you resumed coital activity?”* could be a good screening tool to detect occult postpartum sexual dysfunction related to painful perineal tears which required medical repair. Nevertheless, further prospective studies are needed to provide more in depth knowledge of female sexual function after delivery considering not only physical factors but also social and psychological aspects.

## Data Availability

The data used for these findings were obtained from the clinical history of participating patients. Restrictions apply to the use of these data which are to be used for the current study only and therefore are not publicly available. To obtain access to our raw data the corresponding author (Dr. Cristina Ros-Cerro, Pelvic Floor Unit, ICGON, Hospital Clínic de Barcelona, Spain. E-mail: cros@clinic.cat) should be contacted.

## Abbreviations

AI: Anal incontinence
CS: Caesarean section
ICIQ-UI-SF: International Consultation on Incontinence Questionnaire
MOS: Modified Oxford Grading Scale
OASIS: Obstetric anal sphincter injuries
OVD: Operative vaginal delivery
PISQ-12: Pelvic Organ Prolapse/Urinary Incontinence Sexual Questionnaire-12
SD: Spontaneous delivery
UI: Urinary incontinence
